# Efficacy and safety of radiofrequency ablation for calcified benign thyroid nodules: results of over 5 years' follow-up

**DOI:** 10.1186/s12880-022-00795-5

**Published:** 2022-04-22

**Authors:** Yi Li, Hongying He, Wen Li, Jiahang Zhao, Naiqiao Ge, Yan Zhang, Yukun Luo

**Affiliations:** 1 Department of Ultrasound, Medical School of Chinese PLA, Beijing, China; 2 Department of Ultrasound, the First Medical Centre, Chinese PLA General Hospital, Beijing, China

**Keywords:** Radiofrequency ablation, Moving shot technique, Benign thyroid nodule, Calcification, Volume reduction rate

## Abstract

**Purpose:**

To evaluate the efficacy and safety of radiofrequency ablation (RFA) for treating calcified benign thyroid nodules (CBTNs).

**Methods:**

Fifty-two patients with 52 CBTNs who underwent RFA in our hospital were included in this retrospective study. According to the size of calcifications, CBTNs were divided into two groups: the punctate echogenic foci (PEF) group and macrocalcification group. Moreover, the macrocalcification group was further subdivided into two groups, the strong group and the weak group, based on their morphologic characteristics. After the RFA procedure, routine ultrasound (US) and clinical evaluation were performed at 1, 3, 6 and 12 months postoperatively and every 12 months thereafter.

**Results:**

The mean follow-up time was 68.98 ± 7.68 months (60–87 months), and the 5-year mean volume reduction rate (VRR) after RFA was 92.95%, with a complication rate of 0.6% (3/52). The mean initial volume of the macrocalcification group was significantly larger than that of the PEF group (9.94 ± 24.60 ml vs. 0.23 ± 0.22 ml, respectively; *P* = 0.011). Thus, their VRRs were not comparable between the two groups. However, baseline characteristics did not show statistically significant differences between the strong and weak macrocalcification subgroups. The VRRs of the strong subgroup were significantly lower than those of the weak subgroup at the 3-year, 4-year, and 5-year follow-ups.

**Conclusion:**

RFA was effective and safe for treating CBTNs. Strong macrocalcification was related to the VRR of CBTNs after the RFA procedure.

## Introduction

Calcification frequently occurs in thyroid nodules (TNs), occurring in 19.8–32.1% of TNs [1, 2]. The prevalence of calcification is 8–32% in benign nodules and 26–54% in malignant nodules [3]; the prevalence increases with age and the duration of the presence of nodules [4]. Calcification seems to be more prevalent in malignant nodules than in benign nodules. More specifically, macrocalcifications are more likely to be present in benign nodules than in malignant nodules, while microcalcifications are the opposite. According to ATA, macrocalcifications are a criterion of benignity, whereas microcalcifications in a solid hypoechoic nodule (or in the solid hypoechoic component of a partially cystic nodule) are considered to be at high risk of malignancy (estimated risk of malignancy > 70–90%) [5]. In fact, the number of calcified benign thyroid nodules (CBTNs) is increasing annually due the widespread using of ultrasound (US) and the aging of the population. Thus, demands for CBTN treatment are increasing in clinical practice.

Surgery is known as the standard treatment for benign TNs. However, it has several drawbacks, such as a high risk of complications, general anaesthesia requirements, permanent scar formation, etc., which could diminish the quality of life [6, 7]. Thus, nonsurgical and minimally invasive thermal ablation, such as radiofrequency ablation (RFA), has emerged as an alternative for patients with TNs who are ineligible or refuse surgery [8, 9]. Technically, RFA uses a high-frequency alternating current (200–1200 kHz) to oscillate between the anode and cathode, agitating tissue ions and generating heat of friction [10]. Depending on the electrode structure, RFA can be divided into monopolar and bipolar RFA. In monopolar RFA, the electrode acts as a cathode, and a grounding pad is required to release the electric current that flows through the patient’s body [11]. In bipolar RFA, the electric current is confined to the tip of the electrode, which contains both the anode and cathode [12]. Therefore, bipolar RFA overcomes the disadvantage of monopolar RFA, which may cause skin burns with the grounding pad at the contact area and can be used in patients with pacemakers or during pregnancy [13, 14]. Korkusuz et al. [10] published a study comparing monopolar RFA and bipolar RFA in the treatment of benign TNs and showed that bipolar RFA was superior to monopolar RFA in terms of technical efficacy (volume reduction), feasibility and patient discomfort.

In addition to RFA, there are other potential local thermal ablative procedures, such as laser ablation (LA), high-intensity focused ultrasound (HIFU), and microwave ablation (MWA). LA is an efficient and precise treatment, but a single fibre can only produce a small ablation volume. Although a larger ablation volume can be achieved by using multiple fibres, it is more difficult to manipulate several fibres simultaneously than a single electrode [15]. Consequently, incomplete ablation of the nodule margin may result. HIFU can generate thermal tissue destruction without any skin penetration. Since the ablation volume formed by each sonication is small, multiple HIFU impulses have to reach the target tissue, which results in a long treatment time [16]. MWA can produce a larger ablation volume and has a lower heat-sink effect than the other thermal ablation procedures. However, microwave energy must be transported in coaxial cables that are thicker in diameter than the wires used for RFA, resulting in a larger diameter of the MWA applicator than the RFA electrode [14, 17]. Therefore, the flexibility of MWA treatment is less than that of RFA in treating marginally located nodule tissue.

At present, RFA is the most widely used and thoroughly evaluated thermal ablation procedure [18]. It has been recommended for patients with pressure symptoms or cosmetic concerns by several guidelines and consensuses [19–22]. Nevertheless, few studies have assessed the efficacy and safety of RFA for CBTNs, especially the long-term results. Therefore, the purpose of this study is to evaluate the long-term outcomes of RFA for CBTNs.

## Materials and methods

### Patients

The medical records of all benign TNs patients who underwent RFA in our institution between July 2014 and December 2016 were retrospectively reviewed. Nodules that met the following criteria were included: (1) confirmed as benign lesions by US-guided fine needle aspiration (FNA) and/or core-needle biopsy (CNB) before each RFA; (2) detected with hyperechoic foci within the solid component of a nodule; (3) solid nodule composition ≥ 20%; (4) follow-up time ≥ 5 years; (5) refusal or ineligibility for surgery; (6) serum thyroid hormones, TSH (thyroid stimulating hormone) and calcitonin levels within normal range; and (7) age ≥ 18. The exclusion criteria were as follows: (1) benign results in biopsy but shows sonographic evidence, which suspects malignancy, such as extrathyroidal invasion, lymph node metastasis, or distant metastasis; (2) contralateral vocal cord paralysis; (3) history of neck radioiodine therapy; (4) severe coagulation disorder; and (5) serious heart/respiratory/liver/renal failure dysfunction.

**Fig. 1:**
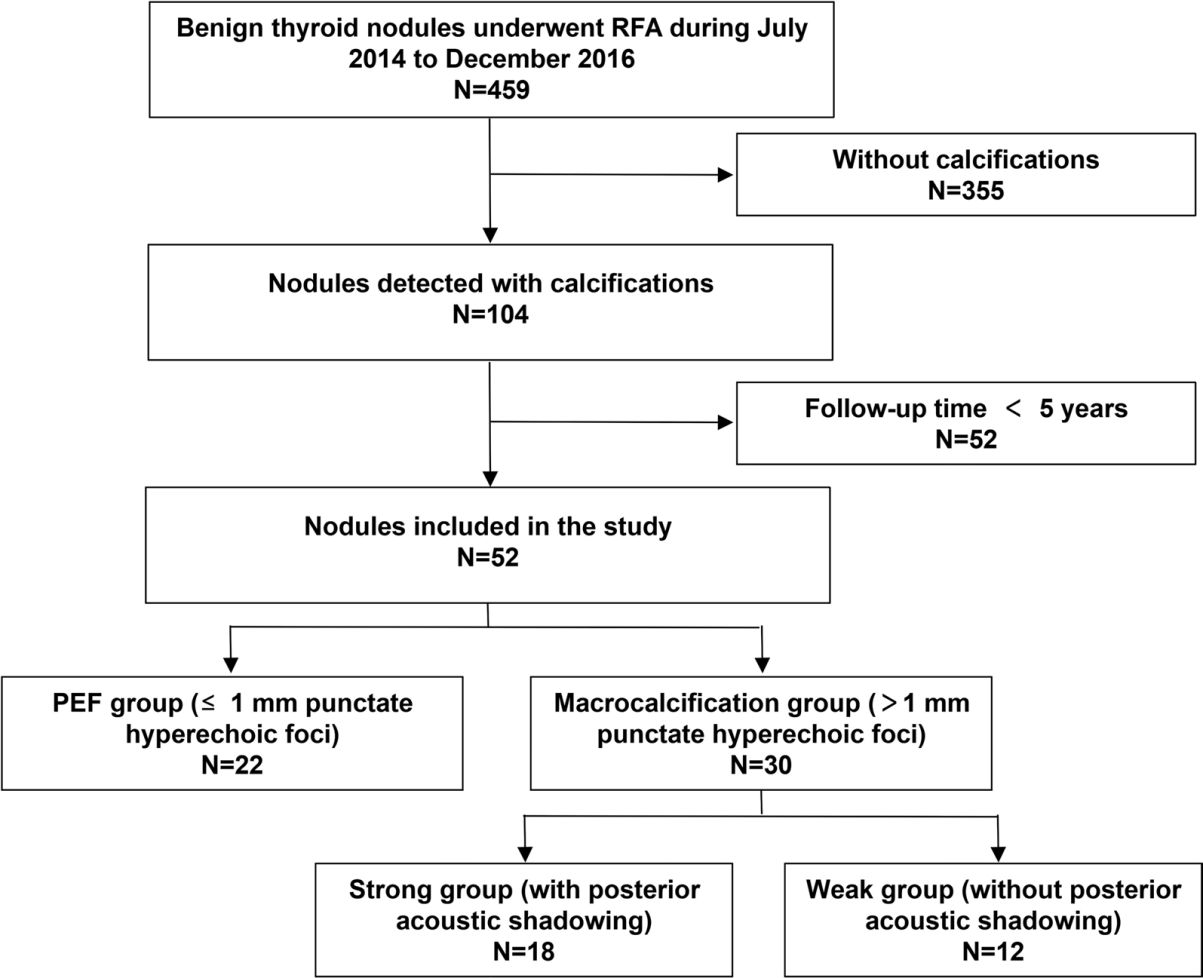
The flowchart of patient enrolment

**Fig. 2:**
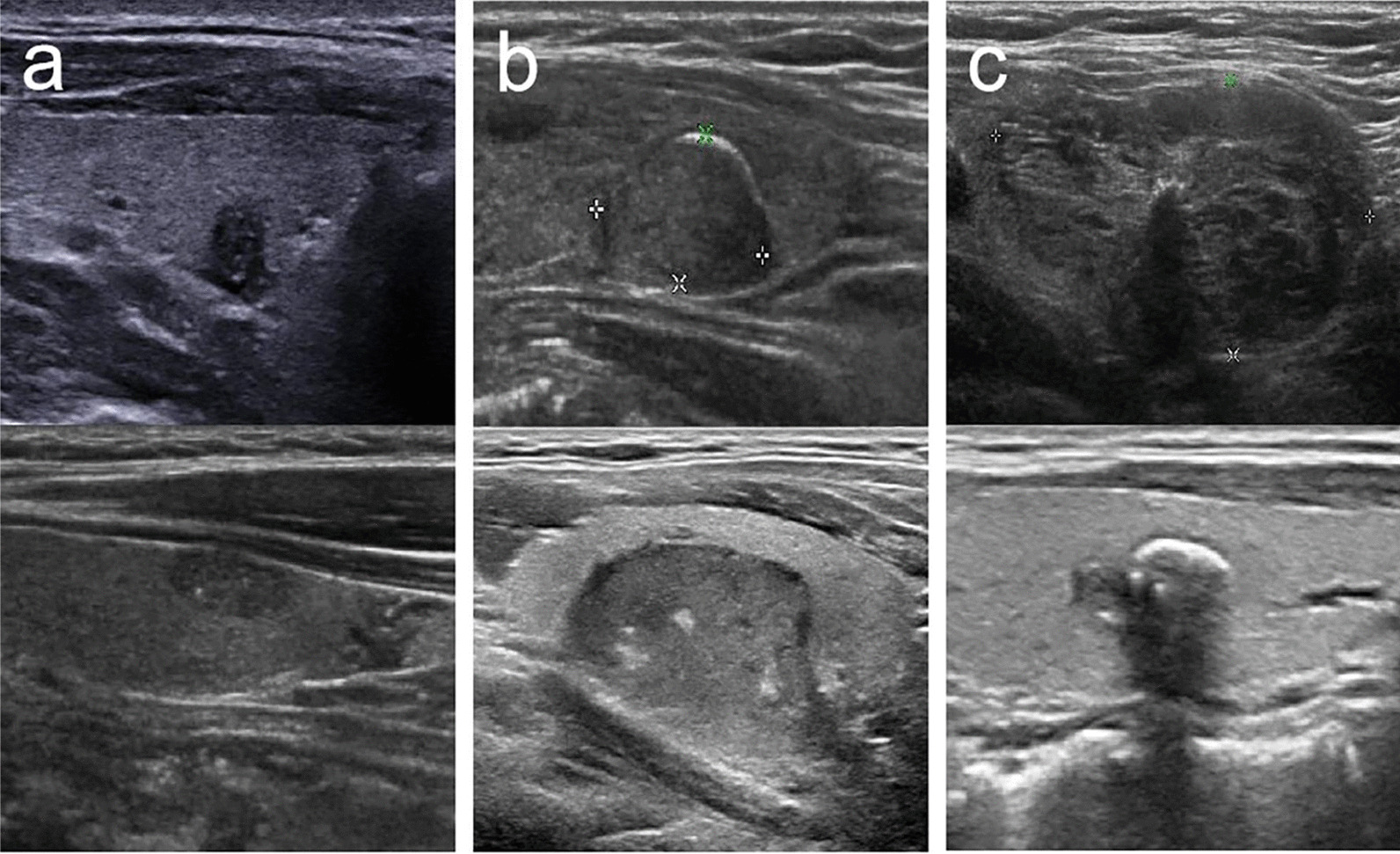
Types of calcifications: **a** PEF, **b** weak macrocalcification, **c** strong macrocalcification

The flowchart of patient enrolment is shown in Fig. 1. Of the 459 benign TNs in the 371 patients identified, 104 nodules were detected with calcifications. Among them, 52 nodules were enrolled in this study, according to the inclusion and exclusion criteria mentioned above. These nodules were divided into two groups: the punctate echogenic foci (PEF) group (≤ 1 mm punctate hyperechoic foci with or without posterior acoustic shadowing) and the macrocalcification group (> 1 mm hyperechoic foci with or without posterior acoustic shadowing), according to the size of calcifications. Moreover, the macrocalcification group was subdivided into two groups: a strong group (with posterior acoustic shadowing) and a weak group (without posterior acoustic shadowing), based on their morphologic characteristics (Fig. 2).

### Preablation evaluation

Prior to treatment, each nodule underwent routine US and contrast-enhanced ultrasound (CEUS). US was performed with Acuson Sequoia 512 (Siemens Healthineers), iU22 (Philips Medical Systems) or M9 (Mindray), and CEUS was performed by injecting 2.4 ml of SonoVue (Bracco International, Italy, Milan) mixed with 5 ml normal saline. Details of nodule size, location, composition, echogenicity, vascularity, and CEUS enhancement degree were recorded. The volume of TNs was approximately calculated by the ellipsoidal formula, which is V = πabc/6 (V is the volume, a is the largest diameter, and b and c are the other two perpendicular diameters).

Nodule vascularity was scored by colour Doppler US as follows [23]: grade 1, no vascularity; grade 2, peripheral nodular vascularity only; grade 3, mild intranodular vascularity (vascularity < 50%), with or without peripheral nodular vascularity; and grade 4, marked intranodular vascularity (vascularity ≥ 50%), with or without peripheral nodular vascularity.

### RFA procedure using moving-shot technology (MST)

All RFA procedures were performed by Y.K.L. (who had over 20 years of interventional US experience) using MST. Patients were placed in a conventionally supine position with a fully extended neck. After sterilization and spreading of sterile towels, local anaesthesia was administered by injecting lidocaine (1%) with an 18-gauge PTC needle. If the nodule contained a cystic component, the PTC needle was inserted into the centre of the cystic area to aspirate as much internal fluid as possible, and anhydrous ethanol injection was repeated (Chang-Hai Hospital, Shanghai, China, G510001). The injection amount of anhydrous ethanol was approximately 50% of the aspirate volume. If not, skip this step. Then, a bipolar electrode with a 9-mm active tip (CelonProSurge, Olympus Surgical Technologies, Germany) was inserted into the target nodule, followed by moving-shot ablation with a 3–7 W output power until the transient hyperechoic echotexture completely covered the target area. For large and/or dense calcifications, ablation was first performed at the periphery of the calcification, and then the electrode was advanced into calcifications for further ablation, slowing down the moving rate of the electrode. The tip of the electrode was visualized in real time with US imaging throughout the procedure to minimize possible complications. CEUS was immediately used to assess the ablation zone: if there was no contrast enhancement in the ablation area (indicating the disappearance of microcirculation in the nodules) [24], the ablation was considered complete; if there was any residual enhancement, complementary ablation was performed.

After RFA, patients were observed for 2 h in the hospital to monitor any signs of discomfort and/or complications. Information for each patient was recorded as follows: duration of application, total amount of applied energy, output power, and major and minor complications. The duration of application was defined as the time between the activation of the needle and the end of the activation of the needle. According to tumour ablation standardization of terminology and reporting criteria [25], major complications were events that could result in mortality, disability or seriously affect the patient’s quality of life (i.e., hypothyroidism, hypoparathyroidism, permanent dysphonia, oesophageal injury, tracheal injury, cervical swelling that presses on the trachea and wound infection), and all others were considered minor complications (i.e., self-limiting dysphonia, cervical pain, localized haematoma, localized swelling, and fever).

### Follow-up

After the RFA procedure, US examinations and clinical evaluations were performed at 1, 3, 6 and 12 months and every 12 months thereafter. Technique efficacy was assessed by the volume reduction rate (VRR), calculated by the following formula: VRR = ([initial volume—final volume] × 100)/initial volume [26]. Nodule regrowth was defined as a ≥ 50% nodule volume increase compared to the minimum recorded volume measured at a given follow-up time point [27–29].

### Statistical analysis

SPSS statistical analysis software (IBM Version 19.0) was used in this study. Continuous data were expressed as the mean ± SD. The Wilcoxon signed rank-sum test was used to compare the initial nodule volumes between the PEF and macrocalcification groups, baseline characteristics (initial nodule volume, energy applied per volume, and vascularity) and VRRs (at each follow-up time point) between the strong and weak macrocalcification subgroups. A t test was used to compare the age between the two macrocalcification subgroups. Categorical data are expressed as frequencies. Fisher’s exact test was used to compare baseline characteristics (sex and location close to critical structure) between the two macrocalcification subgroups. A *p* value < 0.05 was considered significantly different.

## Results

### Baseline characteristics

A total of 52 nodules in 52 patients (39 females, 13 males) were included in this study, and their basic clinical characteristics are presented in Table 1. There were 22 nodules in the PEF group and 30 nodules in the macrocalcification group (12 nodules were in the weak subgroup, and 18 nodules were in the strong subgroup). The CEUS enhancement degree was as follows: hypoenhancement in 28 nodules, isoenhancement in 11 nodules, hyperenhancement in 2 nodules, mixed enhancement in 9 nodules and unidentified enhancement in 2 nodules (could not be assessed due to the strong acoustic shadowing caused by calcification).

**Table 1:** Baseline patients’ characteristics before RFA

Characteristics	Data
Age(years)	45.83 ± 11.12 (18–69)
Sex (F/M)	39/13
No. of patients	52
No. of nodules	52
Initial nodule largest diameter(cm)	1.10 ± 1.18 (0.30–5.80)
Initial nodule volume(cm^3^)	5.83 ± 19.17 (0.02–111.59)
Location	
Left lobe	25
Right lobe	25
Isthmic	2
Location close to critical structures (Y/N)	21/31
Composition	
Solid (fluid component ≤ 10%)	46
Predominantly solid (fluid component 11–50%)	4
Predominantly cystic (fluid component 51–80%)	2
Echogenicity	
Hypoechoic	40
Isoechoic	10
Hyperechoic	2
Vascularity	
Grade 1	22
Grade 2	6
Grade 3	20
Grade 4	4
CEUS enhancement degree	
Hypoenhancement	28
Isoenhancement	11
Hyperenhancement	2
Mixed enhancement	9
Unidentified enhancement	2
Calcification	
PEF	22
Macrocalcification	30
Weak	12
Strong	18

Data are expressed as the mean ± SD (range) or frequency (number of nodules)

### Efficacy

**Fig. 3:**
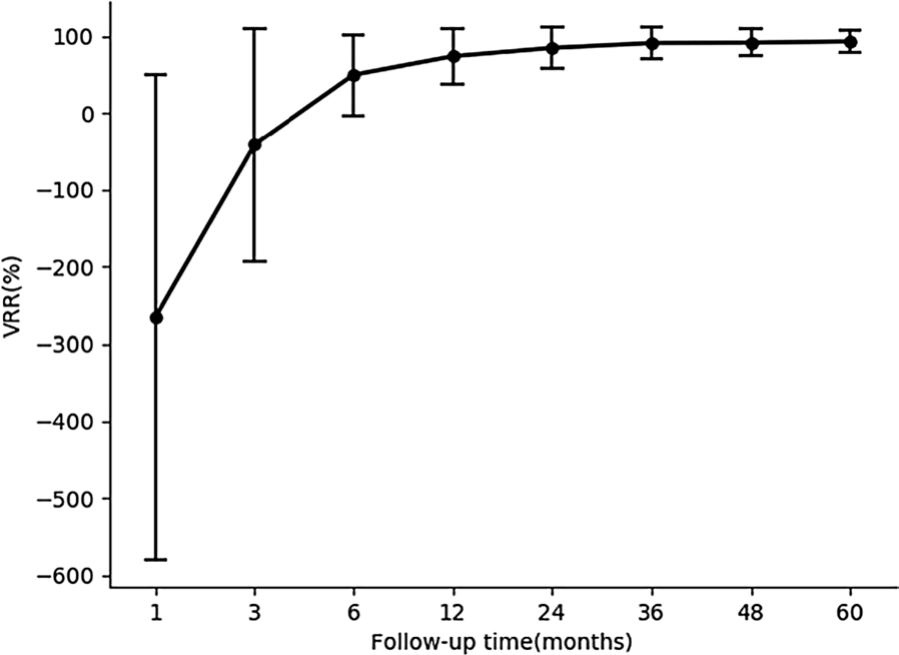
The VRR changes after RFA at each follow-up time point

The mean follow-up time was 68.98 ± 7.68 months (60–87 months). The results of each follow-up time point are presented in Table 2 and Fig. 3. It should be noted that not all patients underwent examinations at all the scheduled time points during follow-up. In all the nodules, secondary ablation was performed in 2 nodules (one was in the weak macrocalcification group and the other was in the strong macrocalcification group), and regrowth was observed in 1 nodule (in the strong macrocalcification group) at the 3-year follow-up.

**Table 2:** The volume and VRR at each follow-up time point after RFA

Follow-up points	Volume (ml)	VRR (%)	Nodules
1 month	2.32 ± 8.92	− 265.35 ± 315.35	32
3 months	1.42 ± 5.29	− 41.28 ± 151.11	27
6 months	1.11 ± 3.81	49.54 ± 52.51	30
12 months	1.09 ± 3.34	73.63 ± 35.55	41
24 months	0.65 ± 2.45	84.53 ± 25.98	41
36 months	0.28 ± 0.93	90.78 ± 19.82	41
48 months	0.25 ± 0.85	91.21 ± 17.43	45
60 months	0.47 ± 1.75	92.95 ± 13.71	52

Data are expressed as the mean ± SD or frequency (number of nodules)

**Fig. 4:**
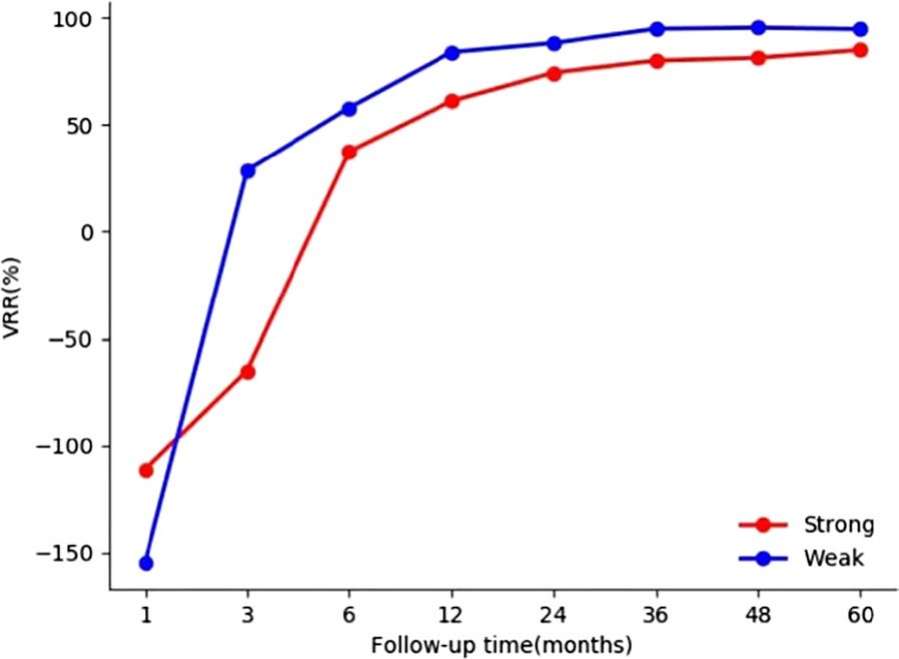
The VRR changes of weak and strong macrocalcification groups after RFA at each follow-up time point

**Fig. 5:**
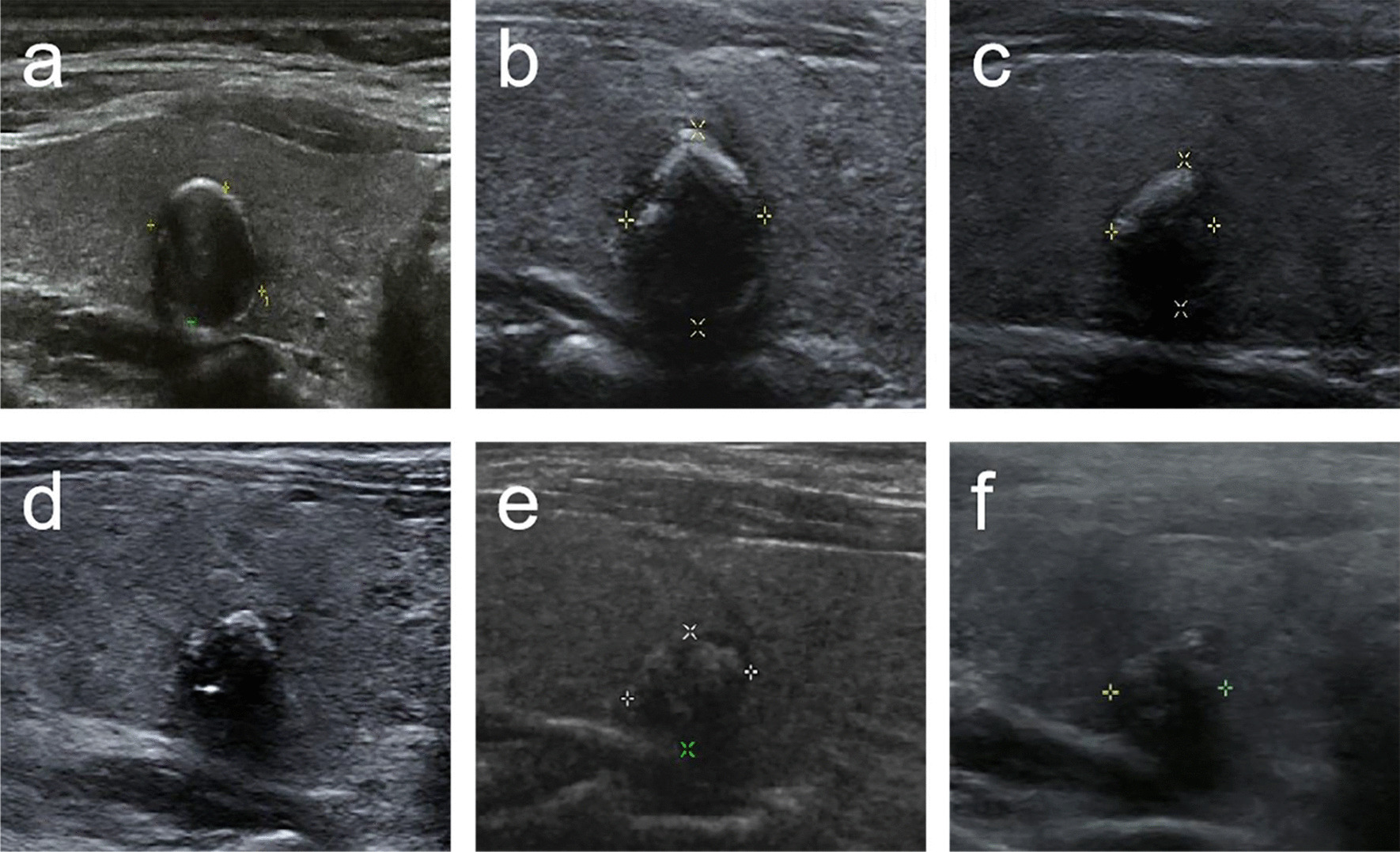
The routine US images of a 45-year-old female in the strong macrocalcification group before ablation and during follow-up: **a** A calcified nodule before RFA with an initial volume of 1.56 ml. **b** At 3 months after RFA, the volume and VRR were 0.65 ml and 58.46%, respectively. **c** At 6 months after RFA, the volume and VRR were 0.39 ml and 74.87%, respectively. **d** At 1 year after RFA, the volume and VRR were 0.32 ml and 79.23%, respectively. **e** At 3 years after RFA, the volume and VRR were 0.39 ml and 74.87%, respectively. **f** At 4 years after RFA, the volume was 0.34 ml, and the VRR was 78.01%

The mean initial volume of the macrocalcification group was significantly larger than that of the PEF group (9.94 ± 24.60 ml vs. 0.23 ± 0.22 ml, respectively; *P* = 0.011). Thus, their VRRs were not comparable. However, the baseline characteristics did not show statistically significant differences between the strong and weak macrocalcification subgroups (all *p* > 0.05), including age, sex, initial volume, energy applied per volume, vascularity and location close to critical structure. The comparative results of macrocalcification subgroups are presented in Table 3 and Fig. 4. The VRRs of the strong subgroup were significantly lower than those of the weak subgroup at the 3-year, 4-year, and 5-year follow-ups (all *p* < 0.05). Routine US images of a representative case are shown in Fig. 5.

**Table 3:** Comparative results of macrocalcification subgroups

Variables	Strong group (n = 18)	Weak group (n = 12)	*P* value (2-tailed)
Age(years)	42.67 ± 10.68	45.08 ± 12.92	0.581
Sex (F/M)	13/5	8/4	1.000
Initial nodule volume(cm^3^)	5.70 ± 14.47	16.29 ± 34.61	0.459
Energy applied per volume (kJ/ml)	4.64 ± 5.93	3.28 ± 4.11	0.472
Vascularity	1.83 ± 0.92	2.50 ± 1.17	0.099
Location close to critical structure (Y/N)	6/12	6/6	0.458
VRR (%)			
1 month	− 111.46 ± 123.44	− 154.94 ± 136.30	0.329
3 months	− 64.85 ± 218.10	28.68 ± 72.71	0.248
6 months	37.49 ± 155.30	57.99 ± 72.06	0.194
12 months	61.15 ± 48.36	83.98 ± 17.14	0.207
24 months	74.42 ± 33.79	88.26 ± 19.97	0.088
36 months	80.05 ± 28.67	94.88 ± 8.79	0.036^a^
48 months	81.32 ± 24.52	95.44 ± 7.73	0.029^a^
60 months	85.03 ± 19.52	94.73 ± 8.97	0.036^a^

Data are expressed as the mean ± SD or frequency (number of nodules)

*p* values were significantly different

^a^Comparison between the weak and strong macrocalcification subgroups

### Safety

The treatment parameters and complications are presented in Table 4. There were no major complications in all patients, but there were only 3 minor complications (cervical pain, localized swelling and electrode fracture). One patient suffered from electrode fracture during the RFA procedure, whose initial nodule volume was 6.72 ml. The VRRs during follow-up were as follows: 60.03% at 6 months, 67.66% at 12 months, 98.12% at 24 months and were maintained until 60 months. However, this patient underwent thyroid right lobectomy for anxiety about malignancy conversion at 5 years after RFA.

**Table 4:** Treatment parameters and complications of RFA

Characteristics	Data
Power (W)	3–7
Duration (s)	299.58 ± 209.71 (47–1371)
Energy (KJ)	1.15 ± 1.09 (0.14–6.72)
Complication	
Major	0
Minor	
Self-limiting dysphonia	0
Cervical pain	1
Localized haematoma	0
Localized swelling	1
Fever	0
Electrode fracture	1

Data are expressed as the mean ± SD (range) or frequency (number of nodules)

## Discussion

In our study, the morbidity of calcification in benign nodules was 22.66% (104/459), which is consistent with previous reports [3, 4]. Fifty-two CBTNs in 52 patients were included and observed over 5 years. The 5-year mean VRR after RFA was 92.95%, with a complication rate of 0.6% (3/52). Based on the aforementioned data, RFA was efficient and safe for treating CBTNs.

At present, the classification standard of thyroid nodule calcifications has not achieved consensus. It is generally classified according to its diameter and morphologic characteristics. In terms of diameter, the thresholds of microcalcifications in US include maximum diameters ≤ 2.0 mm, ≤ 1.0 mm and ≤ 0.5 mm [30], of which ≤ 1.0 mm is common. Meanwhile, macrocalcifications are subdivided into various groups by different researchers based on their morphologic features. Kim et al. [31] classified macrocalcifications into annular, crescent, intranodular and calcified spot subtypes. Kobayashi et al. [32] suggested that macrocalcifications could be subclassified as speckled, fragment, massive, or eggshell. Ha et al. [33] proposed that macrocalcifications could be classified as incomplete (thick and peripheral calcifications in less than 50% of the nodule), complete (thick and peripheral calcifications in more than 50% of the nodule) and rim (thickening that measures less than 0.5 mm with a circumference greater than 50%) subtypes. In this study, a maximum diameter ≤ 1.0 mm was taken as the microcalcification threshold, and macrocalcifications were further divided into strong and weak subgroups based on the presence or absence of posterior acoustic shadowing.

Microcalcification is generally considered to be a marker of papillary thyroid cancer due to its remarkable association with psammoma bodies (PBs). However, US microcalcification is not equivalent to PBs formation, which could also be present in benign nodules. Researchers have demonstrated that microcalcification does not exclusively represent PBs but also other entities, including stromal calcifications, inspissated colloid, puny fibrosis, microcystic area with acoustic enhancement of posterior wall, etc. [30, 34, 35] To avoid misunderstanding, ACR TIRADS recommended a more precise descriptor, “punctate echogenic foci (PEF)”, to replace “microcalcification” [36]. Accordingly, PEF was adopted in this study. There were 22 nodules with PEF in this study. Punctate hyperechoic foci (≤ 1 mm) without comet tail artifact were detected in their solid components, which was considered as suspicious US features [5, 20, 23, 36]. They received initial FNA because of the risk of malignancy. FNA is the first-line diagnostic tool for TNs recommended by ATA [5]. It contains two techniques: one is fine needle aspiration cytology (FNAC); the other is fine needle non-aspiration cytology, that is, fine needle capillary sampling (FNCS). FNCS reduces tissue destruction and bloody specimen by obviating aspiration, resulting in better quality smears [37, 38]. While, FNAC is superior to FNCS in acquisition of adequate tissue sample [37, 38]. However, FNA has higher rates of non-diagnostic and inconclusive results (category I and III in the Bethesda System for Reporting Thyroid Cytopathology) [23]. CNB demonstrated significantly lower rates of non-diagnostic and inconclusive results (5.5% and 8.0%) than those of FNA (22.6% and 40.2%) [39]. In our institution, CNB is used as a repeated diagnostic tool for patients with non-diagnostic and inconclusive results in initial FNA, which has been recommended by consensus and studies [40–42]. The 22 PEF nodules in this study were all diagnosed as benign by FNA or CNB. However, previous studies reported that the false-negative rate of FNA was 2–18%, and that of CNB was 1–3% [43–47]. Furthermore, false-negative rates for FNA and CNB increased to 13.6–56.6% and 4.2–6.2%, respectively, in nodules with suspicious US features [48, 49]. Therefore, these 22 patients with PEF refused active surveillance and underwent RFA for anxiety about the risk of malignancy.

Macrocalcifications have been shown to be associated with FNA failure, which is attributed to inadequate samples; thus, CNB is recommended for nodules with macrocalcifications [33, 50]. In our study, all macrocalcified nodules underwent CNB and obtained diagnostic results before RFA. Furthermore, macrocalcification is considered a relative contraindication for RFA [19] and may have induced RFA treatment failure in a previous study [51]. Unlike liver tumours, TNs are usually elliptical and exophytic and are unsuitable for fixed ablation technique. In 2006, MST was first reported in thyroid nodule treatment by Kim et al. [52] It is a key technique for thyroid nodule RFA, which divides nodules into multiple conceptual ablation units and ablates each unit sequentially by moving the electrode tip [53]. MST may generate an ablation area that conforms to the tumour lesion. Over the past decade, experiences with MST for TNs have shown that it could reduce thyroid nodule volume by 50–85%, with a complication rate of approximately 3% [54–56]. However, calcification in a thyroid nodule may restrict electrode tip movement during RFA [57] and reduce the conduction of heat to the target tissue by altering electrical and thermal conductance [53], which result in insufficient ablation and even treatment failure. Macrocalcified nodules may be challenging in RFA for various reasons, such as its difficulty in penetrating dense calcifications and inability to monitor the electrode tip inside acoustic shadowing. In our study, one patient suffered from electrode fracture when attempting to penetrate dense macrocalcifications. Therefore, the ablation of macrocalcified nodules need more precise and meticulous skill while using the electrode needle.

Factors related to the long-term outcomes of ablation are controversial. Sim et al. [58] summarized that it included nodule-related factors (baseline nodule volume and vascularity) and technology factors. Trimboli et al. [59] reported that the only parameter related to the VRR is the energy delivered by RFA. In this study, significant differences were detected in 3-, 4-, and 5-year VRRs between the strong and weak macrocalcification subgroups. Because the baseline characteristics did not show statistically significant differences between these two subgroups, we suggested that strong macrocalcification is associated with VRR. Fukuoka et al. [60] reported that the cumulative rate of upgrade in the calcification pattern, from weak to strong, was 51.8% at 10 years. Therefore, early ablation of macrocalcified TNs, before the upgrade of weak to strong macrocalcification, may yield higher technique efficacy.

The following are limitations of this study. First, it is a retrospective study with a small sample size. Second, the size of calcification was not quantitatively or semiquantitatively analysed. Third, although data for all patients at the 5-year follow-up time point were completely collected, incomplete follow-up and not performed with a programmed timing may invite important bias. Fourth, all RFA procedures were performed by the same operator, which controlled the influence of operator technique deviation between subgroups comparison. However, it may lead to selection bias and lack of generalizability in other clinical populations. Finally, data were collected from patients who underwent postablation examinations at other hospitals through telephone follow-up, which may introduce bias.

In conclusion, RFA was effective and safe for CBTNs, provided that it is performed by an experienced operator with precise and meticulous skill. Strong macrocalcifications in CBTNs were related to the efficacy of RFA technology. It is necessary to demonstrate whether these findings are reproducible with longer follow-up periods and larger sample size studies.

## Data Availability

Based on patient privacy concerns, the datasets related to the present study are not shared openly. They are available from the corresponding author upon reasonable request.

## Abbreviations

RFA: Radiofrequency ablation
CBTNs: Calcified benign thyroid nodules
PEF: Punctate echogenic foci
US: Ultrasound
VRR: Volume reduction rate
TNs: Thyroid nodules
FNA: Fine needle aspiration
CNB: Core-needle biopsy
CEUS: Contrast-enhanced ultrasonography
MST: Moving-shot technology
PBs: Psammoma bodies
FNAC: Fine needle aspiration cytology
FNCS: Fine needle capillary sampling
